# The traded water footprint of global energy from 2010 to 2018

**DOI:** 10.1038/s41597-020-00795-6

**Published:** 2021-01-11

**Authors:** Christopher M. Chini, Rebecca A. M. Peer

**Affiliations:** 1 Air Force Institute of Technology, Department of Systems Engineering and Management, 2950 Hobson Way, Wright-Patterson AFB, OH 45458 USA; 2 University of Canterbury, Department of Civil and Natural Resources Engineering, Private Bag 4800, Christchurch, 8140 New Zealand

**Keywords:** Energy supply and demand, Energy management, Water resources

## Abstract

The energy-water nexus describes the requirement of water-for-energy and energy-for-water. The consumption of water in the production and generation of energy resources is also deemed virtual water. Pairing the virtual water estimates for energy with international trade data creates a virtual water trade network, facilitating analysis of global water resources management. In this database, we identify the virtual water footprints for the trade of eleven different energy commodities including fossil fuels, biomass, and electricity. Additionally, we provide the necessary scripts for downloading and pairing trade data with the virtual water footprints to create a virtual water trade network. The resulting database contains country-to-country virtual water trade from 2010–2018, broken down by commodity. The purpose of this data descriptor is to provide detailed methods and validation of the dataset beyond the complementary research publication. The resulting database provides opportunities to understand global energy-related water demands and advance future global water resources research.

## Abstract (summary)

**Table:** 

Measurement(s)	water footprint of energy trade • water footprint of electricity
Technology Type(s)	digital curation • data transformation
Factor Type(s)	international trade • electricity generation portfolios • country • year of data collection
Sample Characteristic - Location	global

Machine-accessible metadata file describing the reported data: 10.6084/m9.figshare.13187489

## Background & Summary

Virtual water describes the water consumed in the process of producing a material or good^1^. With respect to energy, water is consumed in the extraction, refining, and production of fuels and in the generation of electricity^2–4^. The energy-water nexus is widely studied, yet primary data regarding energy-related consumption of water resources are scarce. Pairing virtual water estimations with trade data creates a virtual water trade network. Previous instances of virtual trade network estimations have focused on food resources and have spanned global^5–7^, country^8–10^, and city^11–13^ scales. These studies seek to understand the network structure, virtual water savings, and impact on scarce water resources to inform global water and food management.

Recently, similar virtual water studies have been conducted focusing on energy systems, particularly electricity trade. These studies have investigated electricity trade at a continental scale, comparing virtual trade volumes between countries^14^, and at the country-scale with studies in China and the United States. Studies in China often utilize the provinces for nodes (e.g.^15–18^), while studies in the United States have investigated the virtual water trade of electricity at the balancing authority scale^19^. The recent influx of studies, however, generally only focuses on one energy source, electricity, with few exceptions (i.e.^20^). Additionally, many of these studies rely on different methodologies, making comparison challenging.

To that end, here we provide a methodology developed for assessing virtual water footprints and trade associated with energy resources, integrating across multiple databases and previous studies. We identify country-to-country virtual water trade volumes associated with eleven different energy commodities, including coal, hydrocarbons, oil, electricity, and biomass energy sources. As virtual water trade studies of energy systems become more prevalent, this database provides important methods and results to facilitate inter-study comparison and advance our global understanding of transitioning energy systems and associated water impacts. Additionally, through this process, we provide country- and annually-specific water intensity values for energy in exporting countries around the globe. Both the final product of the virtual water trade network and the aggregated water footprint data provide important information for advancing global perspectives of the energy-water nexus and understanding global water concerns. These data are utilized in a related publication detailing the statistics and growth of the virtual water trade network; see Peer and Chini^21^.

## Methods

**Fig. 1:**
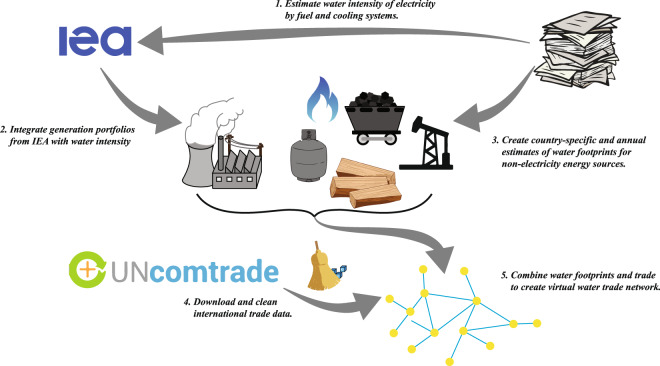
There are five general steps in the creation of the databases, requiring the integration of data from the United Nations, International Energy Agency, and literature sources.

To compute the water footprint of global energy trade, we rely upon data originating from the UN Commodity Trade (UN Comtrade) database, sourced through an Application Program Interface (API) in the R coding language. Trade data are then cleaned to eliminate outliers and erroneous values. The International Energy Agency (IEA) provides information on electricity generation portfolios, retrieved using an API in Python. Water intensity factors are sourced through various literature based on the energy type; see Table 1. Figure 1 illustrates the process of generating the database. Each step is described thoroughly below. All referenced scripts, input files, and output files are accessible via the study’s accompanying Zenodo database^22^, 10.5281/zenodo.3891722. These methods are expanded versions of descriptions in our related work^21^ and offer additional insights to related discussions on water footprints of electricity. This data descriptor facilitates the reproducibility of results and provides the scripts needed to add additional years to the database should the study need to be revisited. Therefore, this data descriptor goes beyond the complementary manuscript by providing greater insights, assumptions, and opportunities on the methods and resultant datasets.

**Table 1:** Water intensity factors and ranges for each energy commodity considered in the database.

Energy	Mean WF (m^3^/kg)	Min WF (m^3^/kg)	Max WF (m^3^/kg)	Source
Coal	3.6 × 10^−4^	0.18 × 10^−4^	4.2 × 10^−3^	^26,28^
Lignite	3.6 × 10^−4^	0.10 × 10^−4^	7.2 × 10^−4^	^26,28^
Crude-Oil	1.1 × 10^−2^	2.0 × 10^−3^	4.8 × 10^−2^	^28,29^
Not-Crude Oil	1.27 × 10^−2^	3.2 × 10^−3^	5.02 × 10^−3^	^28,29^
Natural Gas*	2.14 × 10^−4^	5.34 × 10^−5^	1.44 × 10^−3^	^23,30^
Propane*	2.34 × 10^−5^	5.84 × 10^−6^	1.58 × 10^−4^	^23,30^
Butane*	7.28 × 10^−5^	1.82 × 10^−5^	4.91 × 10^−4^	^23,30^

*Typical, for all countries except China, United States, Argentina, and Canada.

### Determining electricity water footprints

There is no comprehensive database associated with water footprints of electricity across the globe. Therefore, many studies rely on data based in the United States to inform global estimations^23^. For thermoelectric power plants, water footprints vary based on cooling system and fuel type^24^. Water footprint values for thermoelectric power generation were obtained from Macknick *et al*.^3^, a widely utilized reference in the literature. Additionally, Davies *et al*.^25^ provides estimates of cooling technology, globally, by region. The range and expected values of water consumption intensity for each of these generation technologies and fuel types were utilized. Finally, country-specific water footprint values of hydroelectricity were gathered from Mekonnen *et al*.^26^; no uncertainty or range for these values was available. The water intensity values for electricity generation were static and did not vary interannually.

### Creating country-specific water footprints of electricity

Here, we determine the water footprint of electricity for each country based on their electricity generation portfolio. The portfolios are gathered from the IEA for each year^27^. In several instances, specific country values are not available and generation portfolios were manually determined. The database contains the Python script used to interface with the API and download electricity portfolios from 2010-2017 (*IEA-webscraping.ipynb*). At the time of writing, the IEA values were incomplete for 2018 and generation profiles in this year were assumed to be identical to 2017 absent any other data. We also provide the cleaned outputs of this script (*IEA-electricity-mix-20XX-GWh.csv*).

In this study, we consider the water footprint of renewable electricity technologies such as solar or wind power as negligible with respect to its operational stage and assign a water consumption rate of 0 m^3^/MWh to these electricity resources. Utilizing the water intensity factors completed in the previous step, we determine the virtual water footprint of each country, weighted by generation; see Eq. 1. Therefore, interannual variations in a countries’ water footprint are dictated solely by changing electricity generation portfolios.

1 $$VW{F}_{i}=\frac{\sum _{g}{w}_{g}\times {e}_{i,g}}{\sum _{g}{e}_{i,g}}$$

Where *i* is the country of origin, *w* is the water footprint of each electricity generation technology, *g*, and *e* is the electricity generation in each country by technology. This calculation is completed using R and the script, *getElectricityWF.R*. The resultant database contains estimates of water footporint in m^3^/MWh for each country from 2010–2018 (*ElectricityWaterIntensity.csv*).

### Water footprints of other energy sources

#### Fossil fuels

Only static values of water footprint were available for the energy resources of coal, lignite, and oil resources, which did not vary temporally or spatially. Table 1 shows the assumed range of water footprints and the literature sources for these values^23,26,28–30^. All water intensities are provided in m^3^/kg to be consistent with the reported values of the UN Comtrade data. For coal, we assume a conversion value of 36.04 kg/MMBtu to convert between literature estimates. Similarly, we assume a conversion value of 45 MJ/kg for crude oil.

The water consumption of natural gas varies widely depending on the method of extraction. However, there are only four countries that produce shale gas commercially: the United States, Canada, China, and Argentina. Therefore, for exports from these four countries, we define a weighted average of conventional and shale gas extraction water intensity, based on data from the Energy Information Administration^31^. In the process of extracting and processing natural gas, other hydrocarbons such as ethane, propane, butane, or pentanes are produced. Using production factors from the United States, we estimate the ratio of butane and propane production versus natural gas production^32^. Using these ratios, we estimate water footprints of these fuels assuming similar water footprints to natural gas. Approximately 8.1 MMBtu of propane was produced per MMBtu of natural gas from 2010 to 2018. The ratio of butane to natural gas was lower, at 2.6 MMBtu butane per MMBtu natural gas.

#### Biodiesel

Biofuel trade data are only available from 2012–2018. To calculate the temporal and spatial differences in water footprints for biodiesel, we combine water footprint estimates from Gerbens-Leenes *et al*.^33^ and Mekonnen and Hoekstra^34^ with biofuel reports from the US Department of Agriculture (USDA) and Energy Information Administration (EIA) that detail foodstock inputs by weight for biodiesel production. These reports provide a significant amount of data to capture the major countries and foodstock sources, but it is still necessary to create assumptions for the remainder of the countries and foodstocks. Animal fat was a common source for biodiesel production. We estimate that one liter of biodiesel requires 0.88 kg of animal fat and has a yield of 90%^35^, averaging the water footprint of pork, chicken, and beef fat based on global meat production estimates from the UN Food and Agriculture Organization and water footprints of meat^36^. The resultant estimate of water intensity for animal fat-derived biodiesel is 217 m^3^/GJ. Another common component of biodiesel production is used cooking oil, which was assigned a water footprint of zero as it is a waste product. Equation 2 describes the process of generating country-specific biodiesel water footprints.

2 $$W{F}_{C}=\frac{\mathop{\sum }\limits_{C}^{f}{w}_{f}\times {y}_{f}\times {m}_{f}}{\sum {y}_{f}\times {m}_{f}}$$

Where, *C* is a country, *f* is a specific feedstock, *w* is the water intensity of each feedstock, *y* is the yield ratio of each feedstock to biodiesel, and *m* is the mass of feedstock used in each country.

Per the UN trade definition, the biodiesel category must contain less than 70% petroleum based fuels. This creates a wide range of uncertainty in the actual biodiesel content of the trade. To account for this uncertainty, we set mean, maximum, and minimum thresholds of the fuel mix. We assume the mean to be 50% biodiesel, the minimum to be 30% biodiesel, and the maximum to be 70% biodiesel. The resulting water footprints of biodiesel for each exporting country are provided in *BiodieselWF.csv*.

#### Firewood and charcoal

Schyns *et al*.^37^ provide globally gridded blue and green water footprints of roundwood production, attributing the water consumption of forests based on an economic evaluation of the wood product relative to other forest values. Blue water footprints refer to water consumed from surface or groundwater sources, whereas green water footprints are driven by rainfall^38^. The globally gridded values were aggregated by country with minimum, maximum, and average values reported in m^3^_water_/m^3^_wood_. We assume a specific volume of firewood to be 2.08 × 10^−3^ m^3^/kg and 5.92 × 10^−3^ m^3^/kg of charcoal^39^. For countries that did not have gridded values within the dataset, we average values from neighboring countries. No interannual variation in water footprint was available. The final water footprints by country for firewood and charcoal are provided in *FuelwoodWF.csv* and *CharcoalWF.csv*, respectively.

### Trade data download and cleaning

The UN Comtrade data provide the basis for the analysis^40^. These data were downloaded using the *comtradr* package in R, which interfaces with the Comtrade API. Both import and export data were downloaded for all countries from 2010-2018 across eleven different energy commodities. These trade statistics provide the value (USD) of the economic transfer, direction of trade (import or export), trade partners, commodity traded, and the amount of the good transferred. Electricity trade is reported in 1000 kWh (1 MWh); all other energy commodities report trade in kilograms. The script to download trade data is included in the database; see *DownloadComtradeData.R*. Querying the Comtrade API is limited by the number of qualifiers; therefore, the queries are broken down by energy commodity and combined using the *CompileTradeData.R* script provided in the database.

Upon investigation of the data, we identify four areas of data cleaning: (i) resolve differences in imports versus export data, (ii) address discrepancies in electricity trade, (iii) fill data gaps, and (iv) remove outliers.

i To be conservative, in cases where the import and export data were different (or one was not available), the largest traded volume was kept. This assumption was made in absence of any other estimate acknowledging the potential for overestimation. This conservative approach for estimating the water footprint of energy is consistent with similar studies^19^.
ii In some instances, there was reported electricity trade between two non-neighboring countries (i.e., European Countries and the United States). To resolve these potential concerns, we created a database of geographically neighboring countries and inventoried a list of undersea connections and eliminate trades occuring outside these connections. While this assumption would negate potential agreements of electricity trade through countries, we assumed that this proportion of electricity trade is relatively small. Additionally, these assumptions reflect the constraints of electricity trade with infrastructure. Removal of these links is completed in *CompileTradeData.R* using the *ElectricConnection.R* function and the accompanying database of country neighbors, *ElectricityConnections.csv*.
iii For values that were reported as zero but had a monetary value, we determined a unit value ($/kg or $/MWh) as the median of a commodities’ trade between the two countries in the preceding year, following year, and the overall unit value of the commodity originating from the country in the current year. Data gaps are filled using the *filldatagaps.R* script.
iv There were some instances of extreme trade values, particularly in the year 2017. These quantities were often reported two orders of magnitude greater than similar trade links in other years. To manage these errors, we took an objective approach to all reported quantities and identified any values that met all of the following three criteria:

- reported quantities greater than 5 times the median value from other years on the same link,
- reported quantities with a unit value greater than 5 times the median unit value from other years on the same link, and
- reported quantities with a unit value greater than 5 times the median unit value for all exports from the originating country in that year

The above assumptions allowed us to remove extreme values and replace them with estimations based on the reported monetary trade value of the commodity, as above. The script for removing and correcting outliers is provided in the database; see *reviseOutliers.R*.

### Creating the virtual water trade network

Following a cleaned and formatted version of the Comtrade data from Step 4, the virtual water trade network was created by multiplying a water footprint of each energy source, *f*, based on its country of origin *i* and year *y* and the reported trade volume from country *i* to *j*:

3 $$VW{T}_{i,j}^{f,y}={e}_{i,j}^{f,y}\times {w}_{i}^{f,y}$$

**Fig. 2:**
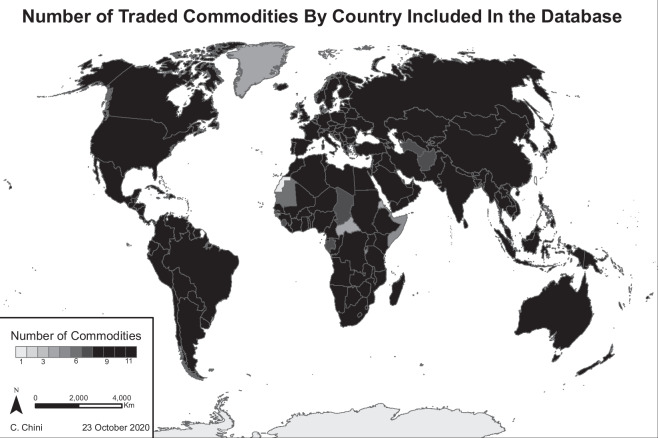
Many countries had export data for all 11 commodities for at least one year from 2010–2018.

For each link in the network, a mean, minimum, and maximum value of trade is calculated based on the ranges of water footprint calculated in Steps 1-3. This step is completed using the *DetermineWF.R* script and results in the main output of the database *EnergyWF_Trade.csv*. The final dataset maintains information on export/import, countries in the trade link, quantity traded, trade value, and associated water footprints. ‘Reporter’ columns refer to the country of origin and ‘partner’ columns are the destination country. The final database includes virtual water exports from 215 countries. Of these countries, only 25 (12%) did not feature exports for all nine years of the analysis. Figure 2 illustrates the global extent of the database with many countries exporting all eleven commodities for at least one year during the study period.

## Data Records

All described data and method scripts are provided in a Zenodo database^22^. The database provides four ZIP files that contain: (i) inputs for determining electricity water footprints of countries, (ii) water footprints of other energy commodities, (iii) all scripts, and (iv) the two final datasets for virtual water trade and water footprints of electricity by country. Published data are available from 2010–2018, except for virtual water trade and water footprints of biodiesel, which is only available in the UN Comtrade database from 2012–2018.

## Technical Validation

The data utilized in this study originate from widely utilized databases and heavily cited literature sources. We therefore validate our datasets through comparison with previously published work.

**Fig. 3:**
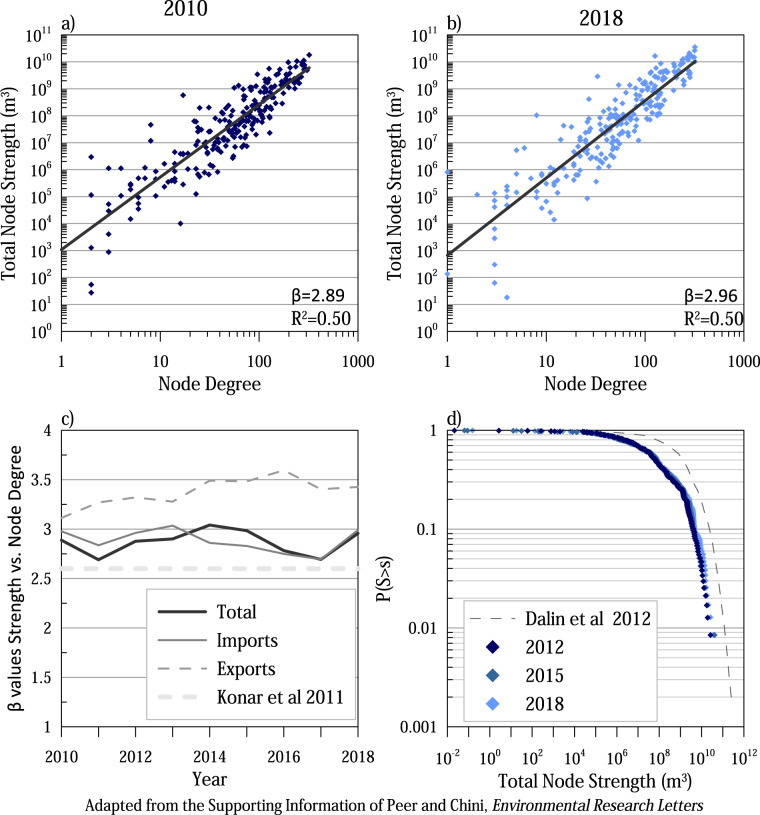
The energy virtual water network structure is similar to food virtual water networks analyzed by Konar *et al*.^5^ and Dalin *et al*.^7^ Figure is adapted from the network statistics in the supporting information of Peer and Chini^21^.

There are multiple similar studies that can be used to validate our results. First, previous research of global virtual water trade networks have identified network structures that can be compared to evaluate the validity of the research. While these previous studies have predominantly focused on food resources, we can reasonably expect similar trade structures in energy trade due to existing trade agreements. Figure 3 illustrates the network properties of strength, node degree, and strength distribution associated with the provided data. Figure 3(a,b) describe the relationship of total node strength to total node degree, illustrating a power-law relationship between the indicators. The relationship remains relatively constant from 2010–2018, with minor variations in the *β* value. Comparing these values to the global virtual water network of food from Konar *et al*.^5^ shows that both networks have a power-law relationship, but the energy network scales slightly higher than food (*β* = 2.6), Fig. 3c. Additionally, Konar *et al*.^6^ break down the food virtual water network into import and export relationships noting differences in the structure of imports and exports. Finally, Dalin *et al*.^7^ determines that node strength distribution for the global food virtual water trade network scales consistent with a stretched exponential fit, plotted on Fig. 3d. The shape of the distribution is similar to node strength distribution presented in this database, with a shift to the right, consistent with higher reported total virtual water trade values associated with food than energy. The underlying network shape suggests that the virtual water trade network of energy follows basic patterns of global trade networks.

**Fig. 4:**
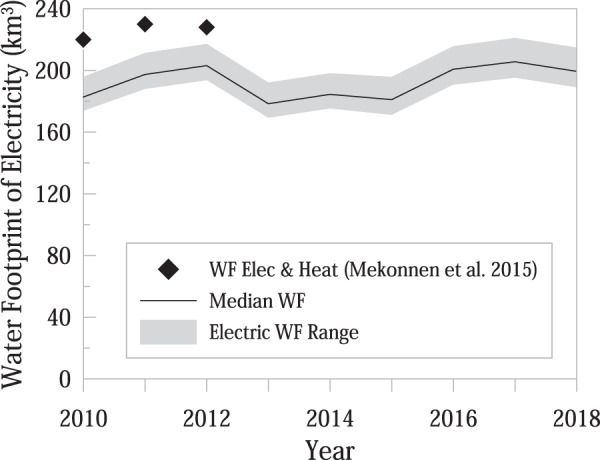
The calculated water footprints of electricity are similar to global estimates provided by Mekonnen *et al*.^26^.

Second, a study by Mekonnen *et al*.^26^ identified the global water footprint of electricity and heat with three overlapping years to the current database (2010–2012). Mekonnen *et al*.^26^ break down the water footprint based on fuel source, including firewood, hydroelectric, and fossil fuels. As the current study only considers electricity and raw energy sources (not heat generation, specifically), we exclude the water footprint of firewood for the comparison as it is not a widely used source for electricity generation. Figure 4 shows the comparison of the values from Mekonnen *et al*.^26^ to our estimates of water footprint for electricity, on the global scale. Estimates by Mekonnen *et al*.^26^ are only marginally greater than the estimates provided in the database (<20%). As the authors also include estimates associated with heat generation, we assert that the current assignment is validated by previously published literature.

## Usage Notes

The complete and cleaned inputs and scripts to create the virtual water trade network and electricity water footprint outputs are provided in the accompanying database. Code and inputs are provided to promote visibility of assumptions and reproducibility of the results. Output files are provided for further use in global energy-water nexus studies. The virtual water trade network is intended to be utilized as one would use the original Comtrade database, to understand flows of materials between countries. Additionally, the methods and assumptions utilized to clean the Comtrade data could be utilized to facilitate the cleaning of other, non-energy commodity trades reported by the United Nations.

## Data Availability

All code necessary for generating the overall virtual water trade network is published in Zenodo^22^, 10.5281/zenodo.3891722. Most of the coding was completed using the R scripting language and open source packages, with one script written using Python to access the IEA API. See Methods for a description and breakdown of use for each script file. Versions and Packages: • R-base: 4.0.0 • comtradr (R): 0.2.2 • Python: 3.7.4 • pandas (Python): 0.25.3 • numpy (Python): 1.18.1 • requests (Python): 2.22.0 • json (Python): 2.0.9
